# Factors contributing to the implementation of interventions to prevent and manage intensive care unit delirium: a systematic review protocol

**DOI:** 10.1136/bmjopen-2024-093338

**Published:** 2025-04-28

**Authors:** Burak Kundakci, Katherine Louise Jones, Andrew Booth, Roxanne M Parslow, Andrew J Moore, Ben Gibbison, Maria Pufulete

**Affiliations:** 1 Sheffield Centre for Health and Related Research (SCHARR), The University of Sheffield, Sheffield, UK; 2 Centre for Musculoskeletal Research, The University of Manchester, Manchester, UK; 3 School of Social and Community Medicine, University of Bristol School of Social and Community Medicine, Bristol, UK; 4 Translational Health Sciences, University of Bristol, Southmead, Bristol, UK; 5 University of Bristol, Bristol, UK

**Keywords:** Delirium, Implementation Science, Intensive Care Units

## Abstract

**Abstract:**

**Introduction:**

Delirium is a common and serious condition that frequently affects patients in the intensive care unit (ICU). It is characterised by an acute disturbance in cognition, attention and awareness that develops over a short period of time and tends to fluctuate in severity. Patients with ICU delirium (ICUD) may experience confusion, disorientation, difficulty focusing and perceptual disturbances such as hallucinations or delusions. The prevalence of ICUD is high, with estimates suggesting that it can affect up to 70% of ICU patients. The development of ICUD is associated with several adverse outcomes, including prolonged ICU and hospital stays, increased healthcare costs, higher mortality rates and an increased risk of long-term cognitive impairment, including dementia. It is unclear which components should be included in a complex intervention to prevent and manage ICUD. Furthermore, we need to understand how the different components have been implemented and their impact on clinical practice.

**Methods and analysis:**

The review will be reported according to the Preferred Reporting Items for Systematic reviews and Meta-Analysis Protocols (PRISMA-P) and the enhancing transparency in reporting the synthesis of qualitative research (ENTREQ) reporting recommendations. We will perform systematic searches to identify relevant interventions and implementation strategies for the prevention or management of ICUD. We will assess primary research, service evaluations and audits for the use of the Standards for QUality Improvement Reporting Excellence (SQUIRE) as a checklist for quality improvement in healthcare. We will extract both qualitative and quantitative data and assess study quality using the Critical Appraisal Skills Programme (CASP) tool. Our findings will be synthesised using a best-fit framework synthesis mapped against the Theoretical Domains Framework (TDF). Our Patient and Public Involvement (PPI) group will contribute to the development of review processes such as the research question and methodology and will help to evaluate which outcomes are most important.

**Ethics and dissemination:**

No ethical approval is required for this study. The results of this systematic review of implementation strategies will be disseminated through peer-reviewed publications and conferences. They will also form part of an evidence map and logic model for factors that can improve the implementation of strategies for prevention, identification and management of ICUD.

**PROSPERO registration number:**

CRD42024537313.

**Strengths and limitations of this study**

- The study employs a mixed methods systematic review incorporating qualitative data using the Theoretical Domains Framework and intervention components from previous quantitative overviews, allowing for a holistic understanding of implementation factors.
- The study protocol outlines a comprehensive search approach including multiple databases, CLUSTER searches for associated qualitative studies, and implementation-specific search filters.
- Quality assurance processes include dual independent reviewer screening, data extraction, and quality assessment using recognised appraisal tools, enhancing the reliability of review findings.
- Patient and Public Involvement (PPI) is incorporated throughout the review process, contributing to the research question, methodology and outcome prioritisation, which strengthens relevance and applicability.
- Limited language and geographical scope: The review is restricted to English-language publications and studies from healthcare systems comparable to the UK (US, Canada, Australia, New Zealand and the European Union), potentially missing relevant implementation experiences from other contexts.

## Introduction

### Description of the condition

 Intensive care unit delirium (ICUD) is a prevalent and serious form of acute brain dysfunction, affecting up to 70% of patients admitted to intensive care.^1^ In the UK, it is estimated that over 171 000 ICU patients experience delirium each year.^1 2^ However, this figure may be an underestimation due to limitations in current diagnostic tools. The incidence of ICUD is expected to rise as the population ages and more individuals with multiple comorbidities require intensive care.

Patients who experience ICUD suffer from cognitive impairments, including difficulty thinking clearly, maintaining attention and comprehending their surroundings. They may also experience perceptual disturbances, such as hallucinations or delusions, which can be highly distressing for both patients and their loved ones. Many factors contribute to the likelihood of developing delirium, including the underlying illness, pre-existing comorbidities, medications used in the ICU (eg, sedatives and analgesics), infections, severe pain, impaired cerebral oxygen utilisation and withdrawal from substances like alcohol and nicotine.

The occurrence of ICUD has significant consequences, including prolonged ICU and hospital stays, with an HR for discharge of 0.65 (95% CI 0.55, 0.76).^3^ This extended length of stay translates to increased healthcare costs, estimated at around £13 000 per hospital stay.^4 5^ Moreover, ICUD is associated with higher mortality rates^3 6^ and an increased risk of long-term cognitive impairment.^7 8^

Recognising the importance of addressing ICUD, various healthcare organisations have prioritised this issue. Assessing patients for delirium was identified as an unmet need in the UK Department of Health and Social Care’s Dementia 2020 Challenge.^9^ Additionally, the National Institute for Health and Care Excellence (NICE) and the Royal College of Physicians have listed ICUD as a high-priority research area.^10^ Furthermore, the James Lind Alliance’s Intensive Care Priority Setting Partnership, which includes input from patients, families, and healthcare professionals, has ranked ICUD among its top three priorities.^11^ Given the significant impact of ICUD on patient outcomes and healthcare systems, efforts to prevent its occurrence and minimise its duration are crucial.

### Interventions used to prevent and treat ICUD

Both pharmacological and non-pharmacological interventions have been used to prevent and manage ICUD. Pharmacological interventions may include avoidance of benzodiazepines, use of dexmedetomidine for sedation,^12^ antipsychotics^13^ and melatonin.^14^ Non-pharmacological interventions may include repeated reorientation of patients, spontaneous awakening trials, sleep protocols and use of a scheduled pain management tool. The optimal intervention is likely to include multiple components, although these have not been adequately defined and agreed by clinicians. The ABCDEF bundle^15^ developed and promoted by the US Society of Critical Care Medicine (SCCM) is one example of a defined complex intervention. It has been found to improve mortality, ICU and hospital stays,^16^ but barriers to its implementation include, for example, patient instability and safety concerns, increased workload, lack of staff’s knowledge, lack of clinician engagement because of perceived lack of efficacy, staff safety concerns, unclear protocol criteria, overly complex protocols and lack of coordination among interprofessional care teams.^17 18^ Implementation is a complex process that is often hindered by individual-level barriers, organisational-level barriers and system-level barriers.^19^

### Why it is important to do this review

To adequately design an effective complex intervention, we need to gather information about the interventions (investigated as part of a separate review,^20^ PROSPERO: CRD42024537313) and their implementation. Implementation strategies have been investigated in a previous review; however, this^21^ did not include qualitative evidence or focus on barriers and facilitators to implementation. We need to understand what factors affect how these interventions are implemented so that we can design a complex intervention that is not only *effective* at preventing and managing ICUD in a research context but can also be implemented successfully and sustained in UK healthcare practice. This mixed methods review will consider how interventions to prevent and manage delirium in the ICU have been implemented and identify the barriers and facilitators to do so.

### Research question

What are the barriers and facilitators to implementing interventions (single or bundles of care) to prevent and manage ICUD?

### Objectives

1. To describe how interventions to prevent and manage ICUD have been implemented.
2. To identify the factors that influence implementation and their impact on clinical practice.

## Methods and analysis

### Design

A mixed methods systematic review of implementation strategies for interventions to prevent or manage ICUD, following guidance for best practice from mixed methods reviews and systematic reviews of qualitative evidence.^22^ Subsequent reporting of the review will be guided by Preferred Reporting Items for Systematic reviews and Meta-Analysis Protocols (PRISMA-P) 2015 statement^23^ and enhancing transparency in reporting the synthesis of qualitative research (ENTREQ)^24^ recommendations.

### Patient and public involvement

Our Patient and Public Involvement (PPI) group will contribute to the development of review processes such as the research question, methodology, and which outcomes are most important from the patient and carer perspective.

### Inclusion criteria

#### Population and context

We will include studies that report on critically ill adults (aged ≥18 years). We define critically ill patients as those treated in a critical care or ICU of any specialty (eg, burn, cardiac, medical, surgical and trauma) or high dependency unit (HDU). We will exclude those studies conducted in other intermediate care units (eg, coronary care units and respiratory high-care units).

#### Phenomena of interest

We will consider studies that investigate the implementation of the interventions (pharmacological or non-pharmacological) to prevent or manage ICUD. This may include single interventions, care packages/bundles or service interventions. Similar interventions may be used to both prevent delirium and manage delirium (as part of secondary prevention) once it has occurred. A list of candidate interventions will be generated through systematic searches and our meta-review of both pharmacological and non-pharmacological interventions. Because we want to identify all the evidence and review it holistically, we will include both types of interventions, but we will distinguish between preventative and management interventions where possible.

#### Types of studies

This review will consider quantitative, qualitative and mixed methods studies, including comparative and non-comparative study designs (eg, controlled studies, cohort studies and before-after studies), service evaluation and audit. To be included in the review, a study will contain a clear description of the implementation process (ie, an explanation of what exactly was done to implement it). Because the review will be used to guide implementation in the UK National Health Service (NHS), we will only include contextually relevant studies. These include studies from 2000 to the present day. Intensive care has changed significantly since the year 2000. The number of ICU beds has increased,^25^ the staffing and technology have improved and intensive care is now a stand-alone specialty in the UK and internationally,^26^ with its own Faculty, training programme and governance structures.^27^ Therefore, including studies of implementation earlier than this may not be applicable to current UK healthcare practice. We will only include studies with healthcare infrastructure and culture comparable to the UK (US, Canada, Australia, New Zealand and the European Union). Included studies will be limited to English language publications.

### Exclusion criteria

We will exclude studies focused on delirium related to alcohol withdrawal and those solely on the validation of delirium screening tools rather than their implementation. Reviews published since 2000 will be excluded but will be checked for relevant included studies. Protocols, conference abstracts, and proceedings will also be excluded, as they are unlikely to provide sufficient detail on implementation context and processes.

#### Search strategy

An information specialist, guided by expert clinicians, prepared the search strategy (see online supplemental file 1 for the full search strategies for all databases), and conducted a series of searches for interventions and implementation strategies: (1) combining intervention label with a search filter of terms related to implementation and (2) CLUSTER searches for associated qualitative and process evaluations for each intervention.^28 29^ We searched the following databases MEDLINE, EMBASE, CINAHL, PsycINFO and Scopus. The literature searches were conducted in September 2024.

#### Study selection

Following deduplication of search results, all identified citations will be imported into Rayyan.^30^ Titles and abstracts will then be screened by two independent reviewers (from BK, KLJ and AB) for assessment against the eligibility criteria for the review. In addition to the search criteria for contextually relevant evidence, the study selection stage will involve applying geographical limits to ensure retrieval of implementation experiences from comparable health systems. Potentially relevant studies will be retrieved in full text and assessed for eligibility by two independent reviewers (from BK, KLJ, AB, BG and MP). Reasons for exclusion of full text studies that do not meet the inclusion criteria will be recorded and reported in the systematic review. Any disagreements that arise between the reviewers at each stage of the study selection process will be resolved through discussion. The results of the search will be reported in full in the final review and presented in a PRISMA flow diagram.^31^

#### Assessment of study quality

Studies selected for retrieval will be checked by two independent reviewers (from BK, KLJ and AB) for methodological rigour based on the reporting, followed by critical appraisal using the Critical Appraisal Skills Programme (CASP) tool,^32^ which consists of structured checklists designed for different study types, including qualitative research, randomised controlled trials and cohort studies. Each checklist contains a series of questions assessing methodological rigour, clarity of study objectives, reliability of findings, and overall relevance to practice. The checklists are divided into three core sections evaluating study validity, results and applicability, with an additional section assessing methodological soundness in updated versions. Rather than assigning numerical scores, reviewers record responses as ‘Yes,’ ‘No’ or ‘Can’t tell,’ with supporting prompts to highlight key issues for consideration. Any disagreements that arise between the reviewers will be resolved through discussion, or with a third reviewer. The results of quality appraisal will be tabulated and reported narratively. All studies, regardless of the results of their methodological quality, will undergo data extraction and synthesis, where possible.

#### Data extraction

Quantitative and qualitative data will be extracted from studies included in the review by two independent reviewers (BK and KLJ) using a customised data extraction form in Excel piloted on at least five studies. The data extracted will include specific details about the population studied, methods, phenomena of interest, context and outcomes of relevance to the review questions, including outcomes for staff, patients and carers, and costs to the health service.

Quantitative data will comprise of data-based outcomes of descriptive and/or inferential statistical tests regarding implementation. In addition, qualitative data will comprise themes and subthemes with corresponding quotes and will be assigned a level of credibility. Any disagreements that arise between the reviewers will be resolved through discussion, or with a third reviewer.

#### Data transformation

Quantitative data will be converted into ‘qualitised data’. Quantitative data on intervention components will be transformed into textual descriptions or narrative interpretations that can be directly mapped to the elements of the review question. This process involves converting statistical results into thematic content that can be initially coded against the Theoretical Domains Framework (TDF), with any uncoded data being addressed through inductive analysis.

Initially themes will be coded against the TDF.^33^ TDF is an evidence-based framework used to identify influences on healthcare professional behaviour related to implementation of evidence-based practices. It was developed by synthesising 33 theories of behaviour and behaviour change into 14 domains (table 1). This framework is particularly useful for analysing barriers and facilitators to implementation in healthcare settings.

**Table 1:** Theoretical Domains Framework (TDF) domains

Domain	Description
Knowledge	Awareness of the existence of something
Skills	Ability or proficiency acquired through practice
Social/professional role and identity	A coherent set of behaviours and displayed personal qualities in a social or work setting
Beliefs about capabilities	Acceptance of the truth, reality or validity about an ability, talent or facility that a person can put to constructive use
Optimism	The confidence that things will happen for the best or that desired goals will be attained
Beliefs about consequences	Acceptance of the truth, reality or validity about outcomes of a behaviour in a given situation
Reinforcement	Increasing the probability of a response by arranging a dependent relationship between the response and a given stimulus
Intentions	A conscious decision to perform a behaviour or a resolve to act in a certain way
Goals	Mental representations of outcomes or end states that an individual wants to achieve
Memory, attention and decision processes	The ability to retain information, focus selectively on aspects of the environment and choose between two or more alternatives
Environmental context and resources	Any circumstance of a person’s situation or environment that discourages or encourages the development of skills and abilities, independence, social competence and adaptive behaviour
Social influences	Those interpersonal processes that can cause individuals to change their thoughts, feelings or behaviours
Emotion	A complex reaction pattern, involving experiential, behavioural and physiological elements, by which the individual attempts to deal with a personally significant matter or event
Behavioural regulation	Anything aimed at managing or changing objectively observed or measured actions

Any data not coded by these themes will be coded inductively. This will involve transformation into textual descriptions or narrative interpretation of the quantitative results so as to map directly to the elements of the review question.

#### Data synthesis and integration

The review will bring together qualitative evidence from the literature searches together with intervention components identified from two companion overviews of pharmacological and non-pharmacological interventions.^20^ The data will be brought together using the matrix approach which is described by Candy^34^ according to the guidance on integration from the Cochrane Qualitative and Implementation Methods Group.^35^

**Figure 1:**
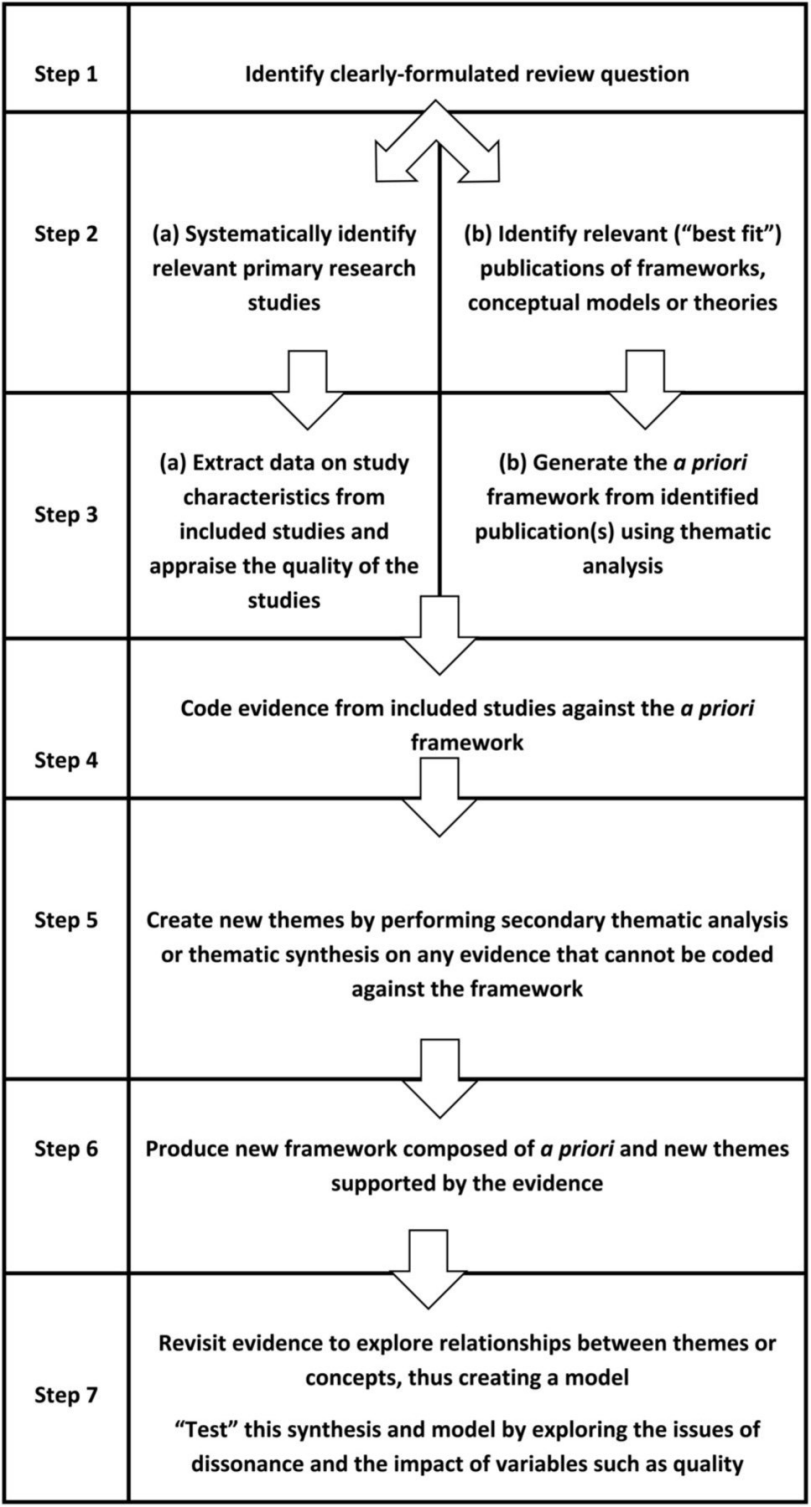
The seven stages of best-fit framework synthesis (from Booth *et al*^36^ 2015).

We will use a best-fit framework *synthesis*^36^ approach that consists of seven stages (figure 1).

Data coding and analysis will be conducted by two independent researchers (BK and KLJ) with expertise in qualitative methods and implementation science. Each coder will initially work separately to code both the transformed quantitative data and qualitative data against the TDF. Weekly consensus meetings will be held to compare coding decisions, resolve discrepancies through discussion, and refine the coding framework. Where consensus on coding cannot be reached a third senior researcher (AB) will provide a summative judgement. For the inductive phase of coding, the team will collaboratively develop new categories through an iterative process as accommodated by best fit framework synthesis, with all members reviewing and validating the final thematic structure. All coding decisions and framework refinements will be documented in an audit trail to ensure transparency and methodological rigour.

The TDF is an implementation framework that provides a theoretical lens through which to view the cognitive, affective, social and environmental influences on healthcare professional behaviour. Best fit framework synthesis methods allow for the addition of an inductive stage, generating new categories or subcategories that may be specific to, or unique to, the context of our study. Best fit framework synthesis methods engineer a distinct separation between the deductive coding phase and the subsequent inductive generation of new labels, allowing readers to identify the unique aspects of the revised framework. Identified barriers and facilitators will be categorised into individual-level, ICU-level and resource-level domains. Where possible, we will explore interactions between different levels of barriers and facilitators.

We will provide full details of intervention components to allow transparent identification of heterogeneity of interventions. We will not be pursuing individual meta-regression of components because this does not allow for synergistic effects from multiple compatible components.

The synthesis will form the basis for an evidence map and logic model for factors contributing to the implementation of interventions to prevent and manage ICUD.

### Stakeholder and PPI review

We have assembled an expert panel of stakeholders, including PPI, intensive care, psychiatric, nursing, physiotherapy and pharmacy clinicians, and researchers with expertise in complex interventions, trial design, qualitative research, statistics, health-services research and psychology to ensure we gather relevant data, identify all implementation methods of the interventions to prevent and manage delirium, and present our findings clearly and comprehensibly.

## Ethics and dissemination

No ethical approval is required for this study. The results of this systematic review of implementation strategies will be disseminated through peer-reviewed publications and conferences. They will also form part of an evidence map and logic model for factors that can improve the implementation of strategies for prevention, identification and management of ICUD.

## Supplementary material

10.1136/bmjopen-2024-093338 online supplemental file 1

## References

[1] Salluh JI, Soares M, Teles JM (2010). Delirium epidemiology in critical care (DECCA): an international study. Crit Care.

[2] Hospital episode statistics: critical care. https://digital.nhs.uk/data-and-information/data-tools-and-services/data-services/hospital-episode-statistics.

[3] Klein Klouwenberg PMC, Zaal IJ, Spitoni C (2014). The attributable mortality of delirium in critically ill patients: prospective cohort study. BMJ.

[4] Varatharaj A, Thomas N, Ellul MA (2020). Neurological and neuropsychiatric complications of COVID-19 in 153 patients: a UK-wide surveillance study. Lancet Psychiatry.

[5] Reade MC, Finfer S (2014). Sedation and delirium in the intensive care unit. N Engl J Med.

[6] Ely EW, Shintani A, Truman B (2004). Delirium as a predictor of mortality in mechanically ventilated patients in the intensive care unit. JAMA.

[7] Girard TD, Thompson JL, Pandharipande PP (2018). Clinical phenotypes of delirium during critical illness and severity of subsequent long-term cognitive impairment: a prospective cohort study. Lancet Respir Med.

[8] Pandharipande PP, Girard TD, Jackson JC (2013). Long-term cognitive impairment after critical illness. N Engl J Med.

[9] Care UDoHaS (2019). Dementia 2020 review: phase 1. https://assets.publishing.service.gov.uk/government/uploads/system/uploads/attachment_data/file/780777/dementia-2020-challenge-2018-review.pdf.

[10] NICE (2019). CG 103: delirium: prevention, diagnosis and management. In: Excellence NIfHaC, ed.

[11] Alliance JL Priority setting partnership: intensive care top 10. http://www.jla.nihr.ac.uk/priority-setting-partnerships/intensive-care/top-10-priorities/.

[12] Hughes CG, Mailloux PT, Devlin JW (2021). Dexmedetomidine or Propofol for Sedation in Mechanically Ventilated Adults with Sepsis. N Engl J Med.

[13] Girard TD, Exline MC, Carson SS (2018). Haloperidol and Ziprasidone for Treatment of Delirium in Critical Illness. N Engl J Med.

[14] Wibrow B, Martinez FE, Myers E (2022). Prophylactic melatonin for delirium in intensive care (Pro-MEDIC): a randomized controlled trial. Intensive Care Med.

[15] Marra A, Ely EW, Pandharipande PP (2017). The ABCDEF Bundle in Critical Care. Crit Care Clin.

[16] Balas MC, Vasilevskis EE, Olsen KM (2014). Effectiveness and safety of the awakening and breathing coordination, delirium monitoring/management, and early exercise/mobility bundle. Crit Care Med.

[17] Morandi A, Piva S, Ely EW (2017). Worldwide Survey of the “Assessing Pain, Both Spontaneous Awakening and Breathing Trials, Choice of Drugs, Delirium Monitoring/Management, Early Exercise/Mobility, and Family Empowerment” (ABCDEF) Bundle. Crit Care Med.

[18] Costa DK, White MR, Ginier E (2017). Identifying Barriers to Delivering the Awakening and Breathing Coordination, Delirium, and Early Exercise/Mobility Bundle to Minimize Adverse Outcomes for Mechanically Ventilated Patients: A Systematic Review. Chest.

[19] Flottorp SA, Oxman AD, Krause J (2013). A checklist for identifying determinants of practice: a systematic review and synthesis of frameworks and taxonomies of factors that prevent or enable improvements in healthcare professional practice. Implement Sci.

[20] Jones KL, Kundakci B, Booth A (2025). Protocol for a meta-review of interventions to prevent and manage ICU delirium. BMJ Open.

[21] Trogrlić Z, van der Jagt M, Bakker J (2015). A systematic review of implementation strategies for assessment, prevention, and management of ICU delirium and their effect on clinical outcomes. Crit Care.

[22] Lizarondo L, Stern C, Carrier J (2019). Mixed methods systematic reviews.

[23] Moher D, Shamseer L, Clarke M (2015). Preferred reporting items for systematic review and meta-analysis protocols (PRISMA-P) 2015 statement. Syst Rev.

[24] Tong A, Flemming K, McInnes E (2012). Enhancing transparency in reporting the synthesis of qualitative research: ENTREQ. BMC Med Res Methodol.

[25] England N Critical care beds sitrep. 2010 - 2023. https://www.england.nhs.uk/statistics/statistical-work-areas/critical-care-capacity/.

[26] ESICM European society of intensive care medicine. https://www.esicm.org.

[27] Nightingale P (2011). Development of the faculty of intensive care medicine. Br J Anaesth.

[28] Booth A, Harris J, Croot E (2013). Towards a methodology for cluster searching to provide conceptual and contextual “richness” for systematic reviews of complex interventions: case study (CLUSTER). BMC Med Res Methodol.

[29] Sutton A, Galvan De La Cruz MC, Leaviss J (2018). Searching for trial protocols: A comparison of methods. Res Synth Methods.

[30] Ouzzani M, Hammady H, Fedorowicz Z (2016). Rayyan-a web and mobile app for systematic reviews. Syst Rev.

[31] Page MJ, McKenzie JE, Bossuyt PM (2021). The PRISMA 2020 statement: an updated guideline for reporting systematic reviews. BMJ.

[32] Singh J (2013). Critical appraisal skills programme. J Pharmacol Pharmacother.

[33] Cane J, O’Connor D, Michie S (2012). Validation of the theoretical domains framework for use in behaviour change and implementation research. Implement Sci.

[34] Candy B, King M, Jones L (2011). Using qualitative synthesis to explore heterogeneity of complex interventions. BMC Med Res Methodol.

[35] Harden A, Thomas J, Cargo M (2018). Cochrane Qualitative and Implementation Methods Group guidance series-paper 5: methods for integrating qualitative and implementation evidence within intervention effectiveness reviews. J Clin Epidemiol.

[36] Booth A, Carroll C (2015). How to build up the actionable knowledge base: the role of “best fit” framework synthesis for studies of improvement in healthcare. *BMJ Qual Saf*.

