# Supplementary material for: Factors contributing to the implementation of interventions to prevent and manage intensive care unit delirium: a systematic review protocol
Source: BMJ Open. 2025 Apr 28;15(4):e093338. doi: 10.1136/bmjopen-2024-093338 (PMC12039013; doi:10.1136/bmjopen-2024-093338)
Supplement: online supplemental file 1 [file bmjopen-15-4-s001.docx]

# Supplemental file 1. Search strategies for all databases

| **Source** | **Search strategy** |
| --- | --- |
| **PubMed** | #18 Search: (Implement[tiab] OR Implemented[tiab] OR Implementation[tiab] OR Implementing[tiab] OR Guideline*[tiab] OR protocol*[tiab] OR "knowledge translation"[tiab] OR Barrier*[tiab] OR Obstacle*[tiab] OR enabler*[tiab] OR Facilitator*[tiab] OR "Process evaluation"[tiab] OR "pilot study"[tiab] OR feedback[tiab] OR "Quality Improvement"[tiab]) OR (improved[ti] OR improving[ti] OR Improvement[ti] OR Improve[ti] OR impact[ti] OR practical[ti] OR practicality[ti] OR practicalities[ti] OR feasible[ti] OR feasibility[ti] OR compliance[ti] OR adherence[ti] OR fidelity[ti] OR multicomponent[ti] OR "multi component"[ti] OR multifaceted[ti] OR "multi faceted"[ti] OR tailored[ti]) AND Delirium AND ("Intensive Care Units" OR "Intensive Care" OR ICU OR ICUs OR "Critical Care") Filters: English, from 2000 - 2024 Sort by: Most Recent |
| **Web Of Science** | S15 ( S8 OR S10 ) AND delirium AND ( ("Intensive Care Units" OR "Intensive Care" OR ICU OR ICUs OR "Critical Care") )  S13 ( S8 AND S10 ) AND delirium AND ( ("Intensive Care Units" OR "Intensive Care" OR ICU OR ICUs OR "Critical Care") )  S12 ( S8 AND S10 ) AND delirium  S11 S8 AND S10  S10 AB ( (Implement OR Implemented OR Implementation OR Implementing OR Guideline* OR protocol* OR "knowledge translation" OR Barrier* OR Obstacle* OR enabler* OR Facilitator* OR "Process evaluation" OR "pilot study" OR feedback OR "Quality Improvement") OR (improved OR improving OR Improvement OR Improve OR impact OR practical OR practicality OR practicalities OR feasible OR feasibility OR compliance OR adherence OR fidelity OR multicomponent OR "multi component" OR multifaceted OR "multi faceted"  Limiters - Publication Date: 20000101-20241231  S8 TI ( (Implement OR Implemented OR Implementation OR Implementing OR Guideline* OR protocol* OR "knowledge translation" OR Barrier* OR Obstacle* OR enabler* OR Facilitator* OR "Process evaluation" OR "pilot study" OR feedback OR "Quality Improvement") OR (improved OR improving OR Improvement OR Improve OR impact OR practical OR practicality OR practicalities OR feasible OR feasibility OR compliance OR adherence OR fidelity OR multicomponent OR "multi component" OR multifaceted OR "multi faceted"  Limiters - Publication Date: 20000101-20241231 |
| **CINAHL** | 1 (Implement or Implemented or Implementation or Implementing or Guideline* or protocol* or "knowledge translation" or Barrier* or Obstacle* or enabler* or Facilitator* or "Process evaluation" or "pilot study" or feedback or "Quality Improvement" or (improved or improving or Improvement or Improve or impact or practical or practicality or practicalities or feasible or feasibility or compliance or adherence or fidelity or multicomponent or "multi component" or multifaceted or "multi faceted")).mp. [mp=title, abstract, heading word, table of contents, key concepts, original title, tests & measures, mesh word]  2 (Implement or Implemented or Implementation or Implementing or Guideline* or protocol* or "knowledge translation" or Barrier* or Obstacle* or enabler* or Facilitator* or "Process evaluation" or "pilot study" or feedback or "Quality Improvement").ab.  3 ("Intensive Care Units" or "Intensive Care" or ICU or ICUs or "Critical Care").af.  4 delirium.af.  5 (Implement or Implemented or Implementation or Implementing or Guideline* or protocol* or "knowledge translation" or Barrier* or Obstacle* or enabler* or Facilitator* or "Process evaluation" or "pilot study" or feedback or "Quality Improvement" or (improved or improving or Improvement or Improve or impact or practical or practicality or practicalities or feasible or feasibility or compliance or adherence or fidelity or multicomponent or "multi component" or multifaceted or "multi faceted")).ti.  6 1 or 2 or 5  7 3 and 4 and 6  8 limit 7 to (english language and yr="2000 - 2024") |
| **EMBASE** | 1 (Implement or Implemented or Implementation or Implementing or Guideline* or protocol* or "knowledge translation" or Barrier* or Obstacle* or enabler* or Facilitator* or "Process evaluation" or "pilot study" or feedback or "Quality Improvement").ab,ti.  2 (improved or improving or Improvement or Improve or impact or practical or practicality or practicalities or feasible or feasibility or compliance or adherence or fidelity or multicomponent or "multi component" or multifaceted or "multi faceted" or tailored).ti.  3 1 or 2  4 delirium.af,kf,kw,ti.  5 3 and 4  6 ("Intensive Care Units" or "Intensive Care" or ICU or ICUs or "Critical Care").af,kf,kw,ti.  7 5 and 6  8 limit 7 to ("remove medline records" and english and yr="2000 - 2025") |
