# Supplementary material for: Factors contributing to the implementation of interventions to prevent and manage intensive care unit delirium: a systematic review protocol
Source: BMJ Open. 2025 Apr 28;15(4):e093338. doi: 10.1136/bmjopen-2024-093338 (PMC12039013; doi:10.1136/bmjopen-2024-093338)
Supplement: Supplementary file 2 [file bmjopen-2024-093338.reviewer_comments.pdf]

PEER REVIEW HISTORY

BMJ Open publishes all reviews undertaken for accepted manuscripts. Reviewers are asked to complete a checklist review form and are provided with free text boxes to elaborate on their assessment. These free text comments are reproduced below.

ARTICLE DETAILS

Title (Provisional)

Factors contributing to the implementation of interventions to prevent and manage intensive care unit delirium: A systematic review protocol

Authors

Kundakci, Burak; Jones, Katherine Louise; Booth, Andrew; Parslow, Roxanne M; Moore, Andrew J; Gibbison, Ben; Pufulete, Maria

---

VERSION 1 - REVIEW

**Reviewer**	**1**
**Name**	**Soni, Kapil**
**Affiliation**	**All India Institute of Medical Sciences, Critical Care**
**Date**	**21-Nov-2024**
**COI**	**None**

I read the systematic review protocol titled *"Factors Contributing to the Implementation of Interventions to Prevent and Manage ICU Delirium"*. Below are my comments and queries:

First, I commend the authors for their efforts to examine the factors influencing the implementation of established strategies for preventing and managing ICU delirium. While our understanding of this complex brain dysfunction has grown significantly over the years, the interplay of its various pathological and physiological mechanisms remains incompletely understood. Despite the availability of evidence-based strategies for prevention and management, their implementation often remains inadequate and inconsistent.

1 - The authors mention that they will include studies addressing barriers to implementing delirium prevention strategies, providing examples such as increased workload and lack of clinician engagement. Could the authors clarify what additional factors they anticipate identifying in the included studies? Providing an a priori supplemental list of potential factors may further enhance the clarity and utility of the protocol for readers.

2 - Do the authors intend to subclassify the identified factors into individual-level, ICU-level (e.g., functioning and culture), and resource-level barriers?

3 - If so, will the review explore potential interactions between these factors? Testing such interactions could provide valuable insights into the multifaceted barriers to implementation.

4 - More clarity is needed on integrating quantitative and qualitative findings. For example, what specific synthesis methods will be applied, and how will these methods be combined to generate actionable insights?

5 - How will the quality of the included quantitative and qualitative studies be assessed? Further details on safeguards for minimizing selection bias during the review process would strengthen the protocol.

6- Given the likelihood of heterogeneity in quantitative studies examining implementation barriers, what strategies will be employed to address this variability?

These clarifications would enhance the scientific rigor of the protocol.

**Reviewer**	**2**
**Name**	**Towell-Barnard, Amanda**
**Affiliation**	**Edith Cowan University**
**Date**	**02-Feb-2025**
**COI**	**None**

Dear Authors,

Thank you for allowing me to review the protocol. I wish you all the best with review.

**Reviewer**	**3**
**Name**	**Krewulak, Karla**
**Affiliation**	**University of Calgary Cumming School of Medicine, Critical Care Medicine**
**Date**	**08-Feb-2025**
**COI**	**None**

Thank you for the opportunity to review this protocol for a systematic review titled “Factors contributing to the implementation of interventions to prevent and manage ICU Delirium.” It’s important to understand what the system- and individual-level facilitators and barriers are to implementing delirium prevention and management strategies in the ICU. The authors provide clear rationale for the systematic review and include a diverse groups of knowledge

users (PPI, clinicians, researchers). Here are a few items that should be considered to ensure transparent reporting of the methods.

1. Page 5, Line 29: Do the authors mean that they will include studies that report on a critically ill adult population?

2. Page 5, Line 44: I wonder if the authors could expand a bit more on what might be a preventative vs. management interventions. For example, would sleep hygiene be preventative or management? Having a few examples or a table that describes them will help others who wish to embark on a related review.

3. Page 5, Lines 45-46: When the authors say "we will also collect additional information on tools/instruments used for screening and diagnosis of ICU delirium," what information do they mean? Per PRISMA-P, please list and define all variables.

4. The TDF framework is well-suited for individual-level facilitators and barriers. Have the authors considered using a framework to address organizational-level facilitators and barriers (e.g., CFIR)? Additionally, including a table describing the TDF would be beneficial.

5. Page 6, Line 34-41: Could the authors add more details about the MMAT, including its scoring and interpretation?

6. Page 6, Lines 59-60: The approach to converting quantitative data into "qualitised data" could be expanded. This might include 1-2 clarifying sentences between the two sentences in Data Transformation. Will the quantitative data be extracted and converted into textual descriptions that are then coded against the TDF?

7. While the data synthesis approach is outlined, the protocol would benefit from more detail regarding the software used and the coding process (per ENTREQ, such as who will be involved with coding and analysis, the procedures for coding, and how coding comparisons will be handled.

8. To enhance transparency for readers, it would be helpful if the authors indicated the page number where each PRISMA-P item is reported versus a Yes/Not applicable.

Thank you again for the opportunity to provide feedback on this important protocol.

VERSION 1 - AUTHOR RESPONSE

Reviewer #1:

I read the systematic review protocol titled *"Factors Contributing to the Implementation of Interventions to Prevent and Manage ICU Delirium"*. Below are my comments and queries:

First, I commend the authors for their efforts to examine the factors influencing the implementation of established strategies for preventing and managing ICU delirium.

While our understanding of this complex brain dysfunction has grown significantly over the years, the interplay of its various pathological and physiological mechanisms remains incompletely understood. Despite the availability of evidence-based strategies for prevention and management, their implementation often remains inadequate and inconsistent.

Author response: Thank you.

1. The authors mention that they will include studies addressing barriers to implementing delirium prevention strategies, providing examples such as increased workload and lack of clinician engagement. Could the authors clarify what additional factors they anticipate identifying in the included studies? Providing an a priori supplemental list of potential factors may further enhance the clarity and utility of the protocol for readers.

Author response: We have now changed our sentence in page 4, lines 124-126 and expanded the anticipated factors based on existing literature.

Action: "…for example, *patient instability and safety concerns*, increased workload, *lack of staff's knowledge,* ~~and~~ lack of clinician engagement because of perceived lack of efficacy, *staff safety concerns, unclear protocol criteria, overly complex protocols, and lack of coordination amongst interprofessional care teams[17,18]."* (page 4, line 124-126)

2. Do the authors intend to subclassify the identified factors into individual-level, ICU-level (e.g., functioning and culture), and resource-level barriers?

Author response: Yes, we will categorise factors into these levels. We have clarified this in the Methods section under the Data Synthesis and Integration heading.

Action: *"Identified barriers and facilitators will be categorised into individual-level, ICU-level, and resource-level domains."* (page 8, lines 282-283)

3. If so, will the review explore potential interactions between these factors? Testing such interactions could provide valuable insights into the multifaceted barriers to implementation.

Author response: While our primary goal is to identify barriers and facilitators, we acknowledge the importance of interactions between these factors. We have now explained this in the Methods section under the Data Synthesis and Integration heading.

Action: "*Where possible, we will explore interactions between different levels of barriers and facilitators.*" (page 8, lines 283-284)

4. More clarity is needed on integrating quantitative and qualitative findings. For example, what specific synthesis methods will be applied, and how will these methods be combined to generate actionable insights?

Author response: We have now clarified this by adding the following paragraph in the Methods section under the Data Synthesis and Integration heading.

Action: "*The review will bring together qualitative evidence from the literature searches together with intervention components identified from two companion overviews of pharmacological and non-pharmacological interventions[20]. The data will be brought together using the matrix approach which is described by Candy[34] according to the guidance on integration from the Cochrane Qualitative and Implementation Methods Group[35].*" (page 8, lines 258-261)

5. How will the quality of the included quantitative and qualitative studies be assessed? Further details on safeguards for minimizing selection bias during the review process would strengthen the protocol.

Author response: After careful consideration, we decided to use the Critical Appraisal Skills Programme (CASP) tool rather than the Mixed Methods Appraisal Tool (MMAT) for assessing the methodological quality of included studies. To minimise selection bias, we will employ a dual independent reviewer process for study selection, data extraction, and quality assessment. Discrepancies will be resolved through discussion or consultation with a third reviewer. We will also document excluded studies along with reasons for exclusion in a PRISMA flow diagram to ensure transparency in the review process. These were already provided in the protocol (Assessment of study quality section), but we appreciate the reviewer's feedback as this section may have been overlooked.

Action: "…followed by critical appraisal using the ~~*MMAT – Mixed Methods Appraisal Tool*[30]~~ *Critical Appraisal Skills Programme (CASP) tool[32], which consists of structured checklists designed for different study types, including qualitative research, randomized controlled trials, and cohort studies. Each checklist contains a series of questions assessing methodological rigour, clarity of study objectives, reliability of findings, and overall relevance to practice. The checklists are divided into three core sections evaluating study validity, results, and applicability, with an additional section assessing methodological soundness in updated versions. Rather than assigning numerical scores, reviewers record responses as 'Yes,' 'No,' or 'Can't tell,' with supporting prompts to highlight key issues for consideration."* (page 6, lines 216-222)

6. Given the likelihood of heterogeneity in quantitative studies examining implementation barriers, what strategies will be employed to address this variability?

Author response: We have now clarified this by adding the following paragraph in the Methods section under the Data Synthesis and Integration heading.

Action: "*We will provide full details of intervention components to allow transparent identification of heterogeneity of interventions. We will not be pursuing individual meta-regression of components because this does not allow for synergistic effects from multiple compatible components.*" (page 8, lines 286-288)

Reviewer #2:
Thank you for allowing me to review the protocol. I wish you all the best with review.

Author response: Thank you.

Reviewer #3:
Thank you for the opportunity to review this protocol for a systematic review titled "Factors contributing to the implementation of interventions to prevent and manage ICU Delirium." It's important to understand what the system- and individual-level facilitators and barriers are to implementing delirium prevention and management strategies in the ICU. The authors provide clear rationale for the systematic review and include a diverse groups of knowledge users (PPI, clinicians, researchers). Here are a few items that should be considered to ensure transparent reporting of the methods.

Author response: Thank you.

1. Page 5, Line 29: Do the authors mean that they will include studies that report on a critically ill adult population?

Author response: Yes, we will include studies that focus on critically ill adults. We have clarified this in the text.

Action: "We will include *studies that report on* critically ill adults (aged ≥18 years).." (page 5, line 161)

2. Page 5, Line 44: I wonder if the authors could expand a bit more on what might be a preventative vs. management interventions. For example, would sleep hygiene be preventative or management? Having a few examples or a table that describes them will help others who wish to embark on a related review.

Author response: As we stated in the introduction, both pharmacological and non-pharmacological interventions have been used to prevent and manage ICU delirium. Many interventions target underlying risk factors and can serve both preventative and management purposes. For example, dexmedetomidine, the ABCDEF bundle, and the use of earplugs and eye masks can be used both to prevent ICUD and to manage existing cases. Given this overlap, there is no strict distinction between preventative and management interventions, as their utility often depends on the clinical context and the stage at which they are applied.

3. Page 5, Lines 45-46: When the authors say "we will also collect additional information on tools/instruments used for screening and diagnosis of ICU delirium," what information do they mean? Per PRISMA-P, please list and define all variables.

Author response: Thank you for highlighting this. As it is not relevant to the main scope of the review, we have deleted that sentence.

Action: "~~*We will also collect additional information on tools/instruments used for screening and diagnosis of ICU delirium."*~~ (page 5, lines 173-174)

4. The TDF framework is well-suited for individual-level facilitators and barriers. Have the authors considered using a framework to address organizational-level facilitators and barriers (e.g., CFIR)? Additionally, including a table describing the TDF would be beneficial.

Author response: We appreciate this suggestion. We will consider the use of the Consolidated Framework for Implementation Research (CFIR) if the extracted data is sufficiently detailed and rich to sustain this. Also, we have added a brief description of the Theoretical Domains Framework (TDF) along with a table outlining its 14 domains.

Action:

"TDF is an evidence-based framework used to identify influences on healthcare professional behaviour related to implementation of evidence-based practices. It was developed by synthesizing 33 theories of behaviour and behaviour change into 14 domains (Table 1). This framework is particularly useful for analysing barriers and facilitators to implementation in healthcare settings." (page 7, lines 245-249)

Table 1. The Theoretical Domains Framework (TDF) Domains

Domain	Description
**Knowledge**	Awareness of the existence of something
**Skills**	Ability or proficiency acquired through practice
**Social/Professional Role and Identity**	A coherent set of behaviours and displayed personal qualities in a social or work setting
**Beliefs about Capabilities**	Acceptance of the truth, reality, or validity about an ability, talent, or facility that a person can put to constructive use
**Optimism**	The confidence that things will happen for the best or that desired goals will be attained
**Beliefs about Consequences**	Acceptance of the truth, reality, or validity about outcomes of a behaviour in a given situation
**Reinforcement**	Increasing the probability of a response by arranging a dependent relationship between the response and a given stimulus
**Intentions**	A conscious decision to perform a behaviour or a resolve to act in a certain way
**Goals**	Mental representations of outcomes or end states that an individual wants to achieve
**Memory, Attention and Decision Processes**	The ability to retain information, focus selectively on aspects of the environment, and choose between two or more alternatives
**Environmental Context and Resources**	Any circumstance of a person's situation or environment that discourages or encourages the development of skills and abilities, independence, social competence, and adaptive behaviour

**Social Influences**	Those interpersonal processes that can cause individuals to change their thoughts, feelings, or behaviours
**Emotion**	A complex reaction pattern, involving experiential, behavioural, and physiological elements, by which the individual attempts to deal with a personally significant matter or event
**Behavioural Regulation**	Anything aimed at managing or changing objectively observed or measured actions

5. Page 6, Line 34-41: Could the authors add more details about the MMAT, including its scoring and interpretation?

Author response: After careful consideration, we decided to use the Critical Appraisal Skills Programme (CASP) tool rather than the Mixed Methods Appraisal Tool (MMAT) for assessing the methodological quality of included studies. We revised the assessment of study quality section in the Methods accordingly.

Action: *"…followed by critical appraisal using the ~~MMAT – Mixed Methods Appraisal Tool[30]~~ Critical Appraisal Skills Programme (CASP) tool[32], which consists of structured checklists designed for different study types, including qualitative research, randomized controlled trials, and cohort studies (REF). Each checklist contains a series of questions assessing methodological rigour, clarity of study objectives, reliability of findings, and overall relevance to practice. The checklists are divided into three core sections evaluating study validity, results, and applicability, with an additional section assessing methodological soundness in updated versions. Rather than assigning numerical scores, reviewers record responses as 'Yes,' 'No,' or 'Can't tell,' with supporting prompts to highlight key issues for consideration."* (page 6, lines 216-222)

6. Page 6, Lines 59-60: The approach to converting quantitative data into "qualitised data" could be expanded. This might include 1-2 clarifying sentences between the two sentences in Data Transformation. Will the quantitative data be extracted and converted into textual descriptions that are then coded against the TDF?

Author response: We have explained this in the Methods section under the Data transformation heading.

Action: "*Quantitative data on intervention components will be transformed into textual descriptions or narrative interpretations that can be directly mapped to the elements of the review question. This process involves converting statistical results into thematic content that can be initially coded against the TDF, with any uncoded data being addressed through inductive analysis."* (page 7, lines 240-243)

7. While the data synthesis approach is outlined, the protocol would benefit from more detail regarding the software used and the coding process (per ENTREQ, such

as who will be involved with coding and analysis, the procedures for coding, and how coding comparisons will be handled.

Author response: We have now clarified this by adding the following paragraph in the Methods section under the Data Synthesis and Integration heading.

Action: "~~We will initially code data against the TDF.~~*Data coding and analysis will be conducted by two independent researchers (BK and KJ) with expertise in qualitative methods and implementation science. Each coder will initially work separately to code both the transformed quantitative data and qualitative data against the TDF. Weekly consensus meetings will be held to compare coding decisions, resolve discrepancies through discussion, and refine the coding framework. Where consensus on coding cannot be reached a third senior researcher (AB) will provide a summative judgement. For the inductive phase of coding, the team will collaboratively develop new categories through an iterative process as accommodated by best fit framework synthesis, with all members reviewing and validating the final thematic structure. All coding decisions and framework refinements will be documented in an audit trail to ensure transparency and methodological rigour."* (page 8, lines 267-275)

8. To enhance transparency for readers, it would be helpful if the authors indicated the page number where each PRISMA-P item is reported versus a Yes/Not applicable.

Author response: We have now provided specific page numbers for each PRISMA-P item.

Action: Updated the PRISMA-P checklist with corresponding page numbers.

VERSION 2 - REVIEW

**Reviewer**	**3**
**Name**	**Krewulak, Karla**
**Affiliation**	**University of Calgary Cumming School of Medicine, Critical Care Medicine**
**Date**	**31-Mar-2025**
**COI**	

Thank you for your careful attention to the feedback. I have no further comments, as the protocol clearly and transparently reports all methods.
